# Examination of heavy metal concentrations and their interaction with anthropogenic sources in Ermenek Dam Lake (Turquoise Lake)

**DOI:** 10.1007/s10653-025-02367-2

**Published:** 2025-01-24

**Authors:** Yusuf Alparslan Argun

**Affiliations:** Kazım Karabekir Vocational School, Waste Management Program, Karamanoğlu Mehmetbey University, Karaman, Turkey

**Keywords:** Heavy metal pollution, Water quality ındex, PCA, HCA, Spatial distribution, Environmental management

## Abstract

This study aims to determine the spatial distribution of heavy metal pollution in Ermenek Dam Lake, water quality assessment and pollution sources. For this purpose, samples were taken 6 times a year from 12 points determined in 2024. Physico-chemical parameters and heavy metals were analyzed in the study. Using the analysis results, indexes such as Water Quality Index and Heavy Metal Pollution Index were calculated. The results show that the lake water complies with TS 266 and WHO standards. The index results indicate that the lake water is at low pollution and risk level and is safe. The distribution and sources of heavy metals were examined using correlation analysis, Principal Component Analysis and Hierarchical Cluster Analysis. Correlation analyses showed that there were significant relationships between pH, temperature, conductivity, dissolved oxygen and TDS and heavy metals. PCA results revealed that Zn was positively correlated with temperature and pH, while Mn and Ni were inversely correlated. The alignment of Pb, Cu and Cd in the same direction showed that these parameters were affected by common sources. HCA results showed that Cr and Fe have similar transport and source properties, while Mn and Ni are affected by different sources. In general, it was determined that heavy metal pollution in lake water was at low levels and local concentrations were present. The study revealed the effects of anthropogenic activities on the lake ecosystem. The findings of the study provide a guiding basis for water quality management in similar hydrological systems.

**Graphical abstract:**

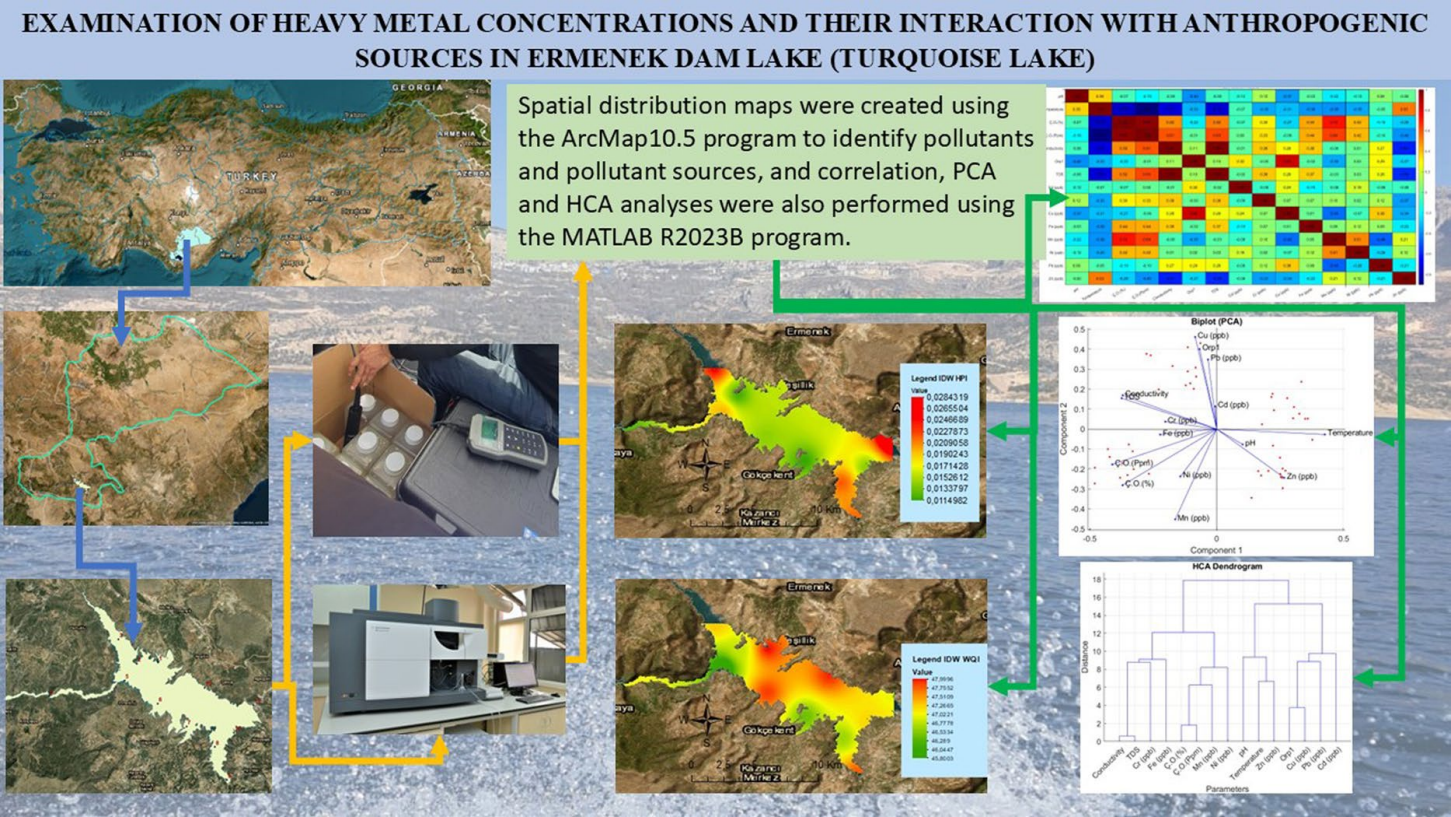

## Introduction

Freshwater ecosystems are facing serious environmental threats due to increasing anthropogenic impacts. Heavy metals, which cause pollution of water resources, have toxic effects on ecosystems and threaten human health. Especially the accumulation of metals such as Cr, Pb, Cd, Zn and Ni in lake ecosystems (Tekin-Özan et al., 2024), which are emitted from urban, industrial and agricultural activities, decreases water quality and threatens biodiversity (Iqbal et al., 2024). Studies on reservoirs in Turkey show that metal concentrations increase especially in summer months and high accumulations are observed in sediments (Yozukmaz & Yabanli, 2023).

International studies show that although heavy metal pollution is influenced by regional, geographical and climatic factors, its impact on lakes poses similar risks and is a global problem (Thirumala et al., 2024; Das, 2024; Pargi et al., 2024; Tu et al., 2024; Raudonytė-Svirbutavičienė et al., 2023). In a study conducted in Karataş Lake, it was observed that heavy metal concentrations increased in autumn and decreased in spring. The concentration distributions of the analyzed heavy metals were classified as Cd < Mo < Cr < Cu < Mn < Zn < Ni < Fe and it was reported that Fe levels exceeded the permitted drinking water standards in all seasons. The Water Quality Index of the lake water was reported to be poor in summer and good in autumn, winter and spring (Tekin-Özan et al., 2024). In the study conducted on heavy metal pollution in Şehriban Creek, it was reported that iron, lead, copper, cadmium, mercury, nickel and zinc accumulations were generally not at dangerous levels for human health, but a slight decrease in water quality was observed from the upper basin to the lower basin. The study stated that water quality was affected by agricultural and geological factors and that there was a seasonal decrease in quality in autumn. According to the results of Water Quality Index (WQI) and Heavy Metal Evaluation Index (HEI), it was emphasized that the stream is in the low contamination category (Tokatlı et al., 2021). In the study conducted on the Kızılırmak River and its coastal environment, it was stated that human activities increased the nutrient, trace metal and other compound loads of the river, which led to environmental pressures and pollution effects on coastal ecosystems. In the study, seasonal changes in water and sediment samples were examined and water quality was evaluated with various quality indices such as sediment quality and trophic status indices. The findings showed that the water quality of the Kızılırmak River was at a moderate level and that the density of algae could increase due to eutrophication in dam areas. In addition, it was reported that lead pollution in water and sediments was caused especially by automobile exhausts and urban runoff (Bakan et al., 2010). In the study conducted on Kainji Dam in Nigeria, Fe and Mn were reported to be present in high average concentrations in water (13 and 9 μg L^−1^), sediment (7092 and 376 μg g^−1^) and fish (11.4 and 4.6 μg g^−1^) samples and were reported to cause toxic effects on aquatic organisms. In the study, Sb, Ti, Cr, Co, Cu and Pb were found to be present in moderate average concentrations in water samples. It was also emphasized that the possible source of the pollutants was anthropogenic and originated from agricultural activities, corrosion/abrasion of ferrous steel material and the dam turbine (Oyewale & Musa, 2006). In the study conducted on Lake Taihu, it was emphasized that the heavy metal concentrations of the surface sediments were ranked as Mn > Ba > Zn > Cr > V > Ni > Pb > Cu > Co, and the content of these nine heavy metals was affected by the geology of the basin and human activities, as well as other factors. According to PCA and PMF analyzes, it was reported that transportation and industrial sources were the most important sources, diagenesis was the second largest source, and agriculture was the third largest source (Yu et al., 2023). In the study conducted in Demirköprü Dam Lake, heavy metal concentrations were measured in surface water, sediment and carp fish (Cyprinus carpio). The results reported that the concentrations of heavy metals (Cd, Cr, Cu, Fe, Ni, Pb) in water samples were generally below national and international standards such as WHO, EC, EPA, TSE-266, SKKY and Irrigation Water Criteria. However, it was stated that especially Fe, Ni and Cu accumulations were high in fish and sediment samples, and this situation could pose a risk to ecosystem and human health (Öztürk et al., 2008). In a study conducted in Lake Gusinoe in Russia, it was determined that the average concentrations of Fe, Zn, Cr, Ni, Cd and Pb in the water did not exceed the Russian national standards and WHO standards. They emphasized that Mn and Cu concentrations exceeded the limit values in winter, spring and autumn due to decomposition in aquatic plants and flow from groundwater sources. It was reported that the index calculation results generally showed low levels of pollution (Bazarzhapov et al., 2023). In a study conducted on rivers feeding Lake Dianchi, it was emphasized that heavy metal pollution was significantly affected by industrial, transportation and agricultural emissions. It drew attention to the accumulation of Cr, Cu, Hg, Zn, Cd and As in the sediments. The pollution load index (PLI) and potential ecological risk index (PERI) calculations emphasized the serious ecological risks posed by these pollutants in the lake ecosystem (He et al., 2024). In the study on heavy metal pollution in Baiyangdian Lake, it was reported that the pollution levels were generally low and in some local areas, moderate pollution caused ecological risks. The study reported that human activities, including agricultural and industrial wastewater, contributed to the accumulation of pollution (Liu et al., 2024a). In the study on lakes in Northern Romania, it was determined that water pollution was primarily caused by industrial and agricultural practices, Fe, Al, Mn and NH4 + . The heavy metal pollution index varied between lakes with average scores ranging from 2.9 to 222, and potential health risks were emphasized (Dippong et al., 2024). In the study conducted on the effects of heavy metals on the environment and human health, it was stated that these metals accumulate in the environment and cause serious damage to both ecosystems and human health. In the study, it was emphasized that agricultural activities and industrial discharges are the main sources of heavy metal pollution and that these metals damage soil, water and vegetation. In addition, it was stated that these pollutants enter the food chain and cause serious health problems such as cancer, neurological disorders and organ damage in humans (Tırınk & Özkoç, 2021).

In this study, the concentrations of heavy metals (Cadmium (Cd), Chromium (Cr), Copper (Cu), Iron (Fe), Manganese (Mn), Nickel (Ni), Lead (Pb) and Zinc (Zn)) and physicochemical parameters (pH, temperature, dissolved oxygen, ORP, TDS, Conductivity) in Ermenek Dam Lake were analyzed and compared with international standards. In addition, pollution sources were identified by using index calculations and clustering methods. This study is a guide for surface water resources with similar geographical, climatic and settlement structures.

## Material and method

### Material

Ermenek Dam is a dam lake built for energy production on Göksu River in Ermenek district of Karaman province. The dam has a concrete arch body type, a body volume of 272,000 m^3^, and a height of 210 m from the river bed. The dam has a lake volume of 4,582.00 hm^3^ at normal water level and a lake area of 58.74 km^2^. The dam, which has an annual power generation capacity of 306 MW and 1,048 GWh, has geographical coordinates determined as 36°34′5″N 32°58′4″E (Çevlik, 2013).

**Fig. 1:**
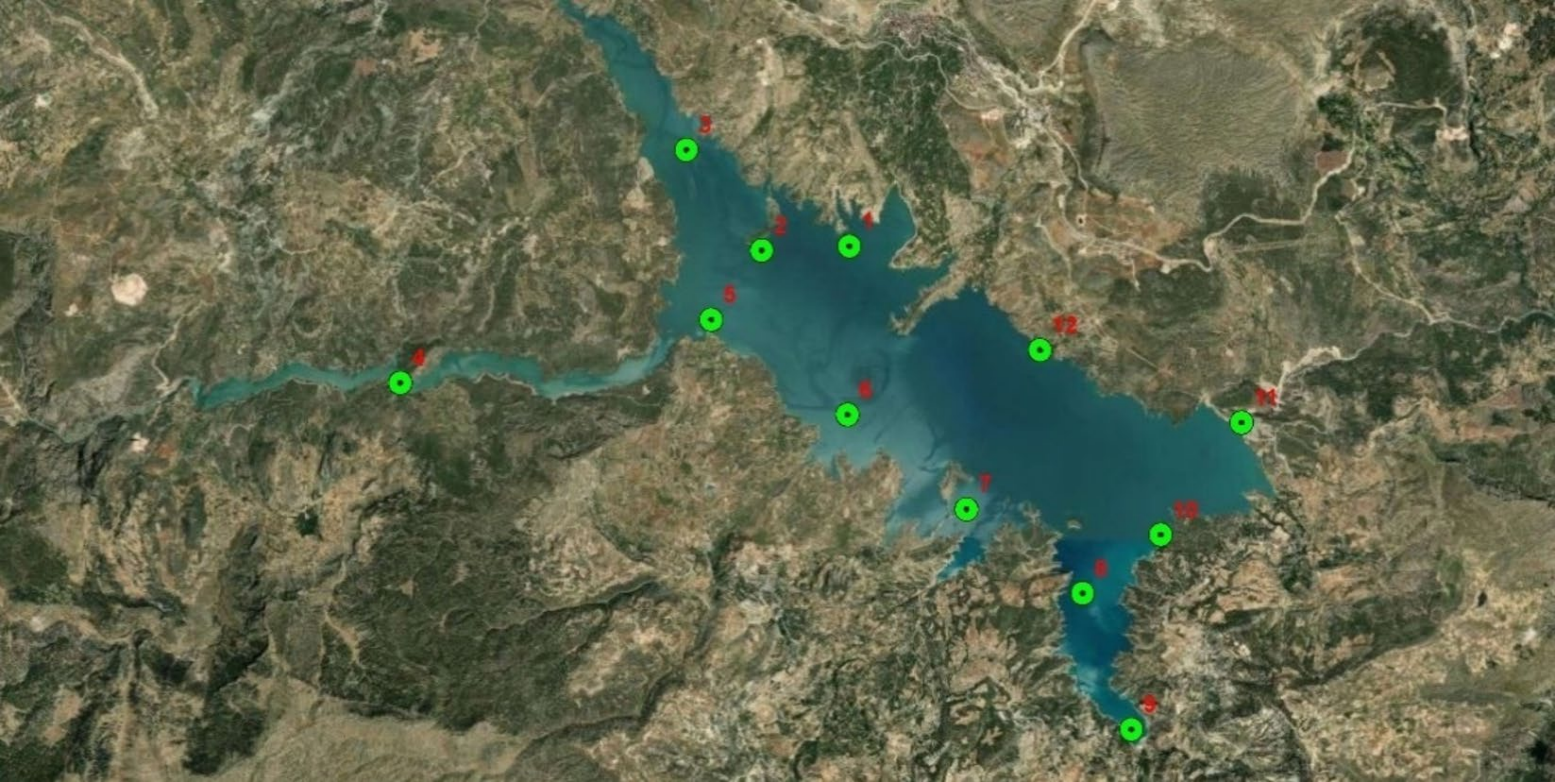
Sample collection points

In the study, in order to determine the water quality of the dam lake, a total of six samples were taken once a month between May and October 2024. Sampling operations were carried out from a total of 12 different points, including the water inlets feeding the dam lake and the inner parts of the lake. The selection of sampling points was made to comprehensively represent the physicochemical properties and pollution distribution of the lake. The sampling points are shown in Fig. 1. There is a wastewater treatment plant discharge point close to sampling point number 1 and a mining (coal) operation and old wild waste storage area close to sampling point number 3. Sampling points number 3, 4 and 9 are on the main water sources feeding the lake. There are village settlements around the lake. The main sources of income of the region are agriculture and animal husbandry. There is no serious industrial activity close to the region.

## Method

### Sample collection process

Samples were taken from the lake with the help of a boat. During each sampling process, water samples taken from a depth of 30 cm below the surface were used in accordance with TS ISO 5667–4 (2019) standards. In each sample taken, the parameters that needed to be analyzed on site were measured first, and then the samples were transported to the laboratory for heavy metal analysis.

### Measurements made in the field

In each sampling process, pH, Water temperature (°C), Dissolved oxygen (DO) (% and mg/L), Electrical conductivity (µS/cm), Oxidation–Reduction Potential (ORP), Total Dissolved Solids (TDS, mg/L) were measured using Hanna HI98194 multi-parameter measuring device.

These measurements were carried out in accordance with ISO 10523:2008 for pH, ISO 7888:1985 for temperature, ISO 5814:2012 for dissolved oxygen (DO), ISO 7888:1985 for electrical conductivity, ISO 11476:1994 for oxidation–reduction potential (ORP) and ISO 10304–1:2007 for total dissolved solids (TDS) standards and recorded in the field. Field measurements played a critical role in collecting instantaneous data on the water quality of the lake (Anderson et al., 2022; Erin et al., 2023; Yudo et al., 2023).

### Heavy metal analyses

The measurement of As, Cr, Cd, Pb, Cu, Ni, B, Fe, Mn, and Zn concentrations in the samples were carried out by the ICP-OES (Inductively Coupled Plasma Optical Emission Spectrometry) method (Douvris et al., 2023). This method provides the measurement of metals in water samples by optical emission spectroscopy and was carried out in accordance with the TS EN ISO 11885 standard.

## Data analysis

### Pollution spatial distribution map

Heavy metal density maps of the lake were created using ArcMap 10.5 software. In ArcMap 10.5 software, the sample points taken from the lake were added to the map according to their geographical coordinates. Heavy metal concentrations measured at each sample point were entered into the attribute table. Spatial density distribution maps were created for each metal with the IDW method using the Interpolation tool from Spatial Analyst tools. IDW calculates the weighted average of the values obtained from known points and enables the estimation of the values at unknown points. In this method, areas close to the measurement points are interpolated to have a higher weight. Thus, a density map of metal concentrations in the lake is created (Liu et al., 2024b; Sun et al., 2024). The spatial density distribution analysis showed in which regions the heavy metal accumulations in the lake are more concentrated and provided important information about the geographical locations of potential pollution sources. These maps are considered as a critical tool for pollution control and management (Wagner et al., 2023; Zhao et al., 2024).

### Correlation and statistical analyses

Correlation, PCA (Principal Component Analysis), HCA (Hierarchical Cluster Analysis) and statistical analyses were performed using MATLAB R2023b software. In correlation analysis, Pearson correlation coefficient was used to evaluate the relationships between heavy metals and each other and with physicochemical parameters. PCA (Principal Component Analysis) was used to identify, recognise and classify the pollution source relationship between the data (Li et al., 2021; Wang et al., 2023). HCA (Hierarchical Cluster Analysis) was applied to examine the similarities of the parameters obtained from the sample points, to evaluate the pollution profiles in different regions and to evaluate the similarities of pollution sources by detecting clusters between parameters (Linnik et al., 2022). This process makes an important contribution to the ecological health of the lake and the identification of the sources of heavy metal pollution (Gao et al., 2020). The results of the analyses were interpreted by comparing them with national legislation and international standards.

### Index calculations and evaluation criteria

In terms of heavy metal pollution of water quality, Water Quality Index (WQI) Eq. 1 (Horton, 1965), Heavy Metal Pollution Index (HPI) Eq. 2 (Reza & Singh, 2010), Heavy Metal Evaluation Index (HEI) Eq. 3 (Edet & Offiong, 2002), Hazard Quotient (HQ) Eq. 4 (Means, 1989), Ecological Risk Index (RI) Eq. 5 (Hakanson, 1980), Comprehensive Pollution Index (CPI) Eq. 6 (Backman et al., 1998) were used in the calculations.

1 $$\text{WQI}=\sum \left(Wi\cdot Qi\right)$$

Here, Wi: is the weight of the parameter $\left(Wi=\frac{1}{Max \,Value}\right)$

Qi: It is the quality ratio of the parameter $\left(Qi=\frac{Measured \,Value-Min.Value}{Max. \,Value-Min.\, Value}\times 100\right)$

2 $$\text{HPI}=\frac{\sum \left(Wi\cdot\, Qi\right)}{\sum \left(Wi\right)}$$

3 $$\text{HEI}=\sum \left(\frac{Measured \,Value}{Max\,Value}\right)$$

4 $$HQ=\frac{Measured \,Value}{Reference \,Dose(RfD)}$$

Here, HQ evaluates the potential effects of each parameter on human health. Reference doses (RfD) are from the EPA.

5 $$\text{RI}=\sum (Tr\cdot \frac{C}{\text{Si}})$$

Here, Tr (Toxicity Factor): Environmental toxicity factor of each parameter. C (Concentration): Measured value of each parameter (mg/L). Si: Standard limit value (mg/L).

6 $$CPI= \frac{\sum \left(\frac{Measured \,Value}{Standard \,Max.Value}\right)}{N}$$

Here, Measured Value: The analyzed concentration of the parameter (mg/L). Standard Maximum Value: The maximum allowed value of the parameter. N: The number of parameters included in the calculation.

In the index calculations and in the evaluation of the analysis results, the standard values given in Table 1 were used. In the evaluation of the findings; the range values for WQI used to evaluate the general quality of water are given in Table 2 (Horton, 1965). The range values for HPI used to evaluate the pollution load of heavy metals in water are given in Table 3 (Prasad & Bose, 2001). The range values for HEI used to evaluate the effects of heavy metals on human health are given in Table 4 (Edet & Offiong, 2002). The range values for HQ used to evaluate the risks to human health are given in Table 5 (US EPA, 1989). The range values for RI used to evaluate the environmental toxicity of heavy metals are given in Table 6 (Hakanson, 1980). The range values for CPI used to determine the general pollution level of water are given in Table 7 (Backman, 1998).

**Table 1:** Water quality parameters, maximum allowable values and health reference limits

Parameter	Unit	TS 266 Standard (TS, 2005)	WHO Standard (WHO, 2017)	Toxicity Factor (Tr) (Hakanson, 1980)	Reference Dose (RfD) (EPA, 1989)
pH	–	6.5–9.2	6.5–8.5	–	–
Temperature	°C	12–25	–	–	–
Dissolved Oxygen	µg/L	≥ 8000	≥ 6000 (min.)	–	–
Conductivity	µS/cm	≤ 2500	≤ 1500	–	–
TDS	µg/L	≤ 1.5 × 10^6^	≤ 10^6^	–	–
ORP	mV	–	–	–	–
Arsenic (As)	µg/L	≤ 10	≤ 10	10	0.3 µg/kg/day
Boron (B)	µg /L	≤ 1000	≤ 2400	1	200 µg/kg/day
Cadmium (Cd)	µg/L	≤ 5	≤ 3	30	0.5 µg/kg/day
Chromium (Cr)	µg/L	≤ 50	≤ 50	2	3 µg/kg/day
Copper (Cu)	µg /L	≤ 2000	≤ 2000	5	40 µg/kg/day
Iron (Fe)	µg/L	≤ 200	≤ 300	1	700 µg/kg/day
Manganese (Mn)	µg/L	≤ 50	≤ 400	1	140 µg/kg/day
Nickel (Ni)	µg/L	≤ 20	≤ 70	5	20 µg/kg/day
Lead (Pb)	µg/L	≤ 10	≤ 10	5	1.4 µg/kg/day
Zinc (Zn)	µg/L	≤ 5000	≤ 3000	1	300 µg/kg/day

**Table 2:** Range values for WQI assessment

WQI range	Interpretation
0–25	Excellent water
26–50	Good water
51–75	Moderately polluted water
76–100	Poor water
> 100	Very poor, unsuitable for use

**Table 3:** Range values for HPI assessment

HPI range	Interpretation
0–15	Low pollution
16–30	Moderate pollution
31–60	High pollution
> 60	Very high pollution

**Table 4:** Range values for HEI assessment

HEI range	Interpretation
< 10	Safe
10–20	Moderate risk
> 20	High risk

**Table 5:** Range values for HQ assessment

HQ range	Interpretation
< 1	Acceptable risk
≥ 1	Health risk

**Table 6:** Range values for RI assessment

RI range	Interpretation
< 150	Low ecological risk
150–300	Medium ecological risk
> 300	High ecological risk

**Table 7:** Range values for CPI assessment

CPI range	Interpretation
< 1	Fresh water
1–2	Moderately polluted water
> 2	Severely polluted water

## Results and discussion

**Fig. 2:**
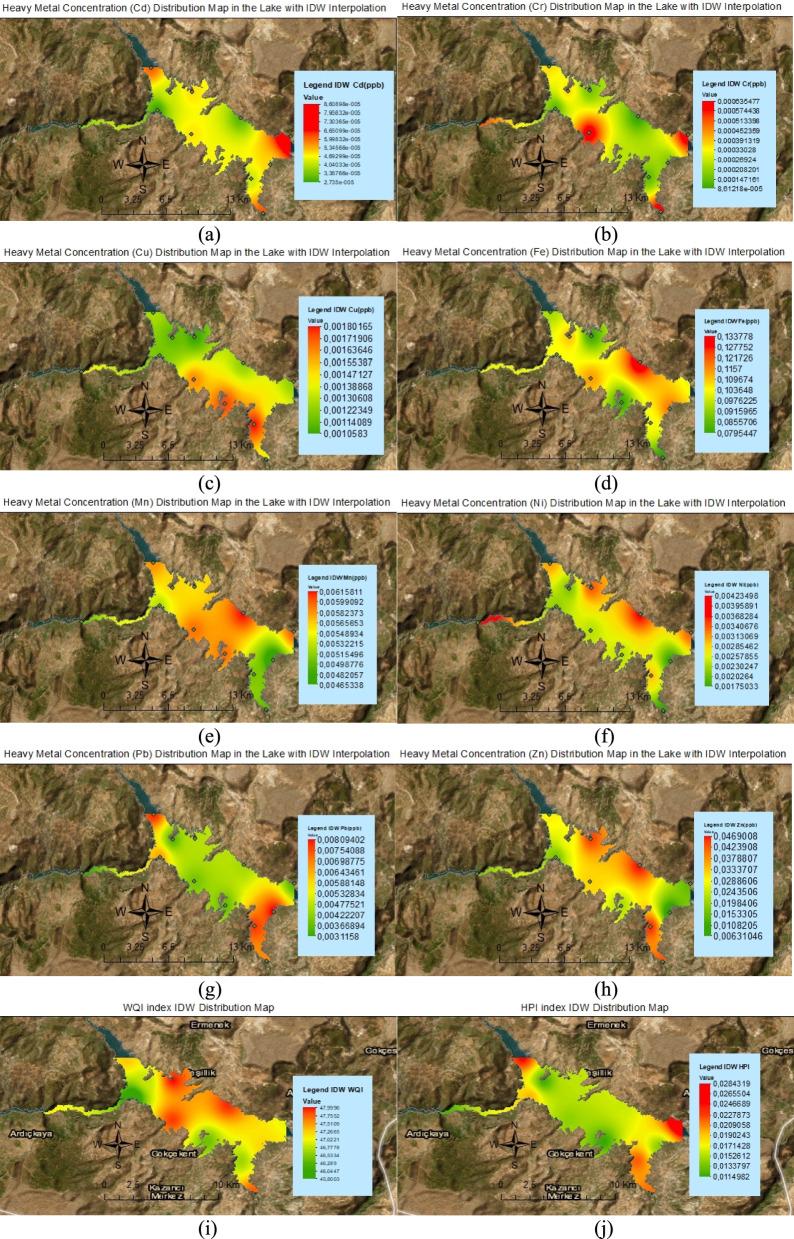
Spatial distribution map of heavy metals in the lake with IDW interpolation; (**a** Cd, **b** Cr, **c** Cu, **d** Fe, **e** Mn, **f** Ni, **g** Pb, **h** Zn, **i** WQI, **j** HPI)

The results of the physicochemical and heavy metal analysis conducted in Ermenek Dam Lake show that the water quality is generally safe and good. As a result of the heavy metal analysis, As and B elements were not detected in the dam lake water. This shows that the metals in question in the analyzed water samples were below the ICP-OES measurement limit. The analysis results regarding water quality and heavy metal pollution are given in Table 8 and the spatial distribution maps of the pollution are given in Fig. 2. In this context, the spatial distribution maps reveal both the distribution of As, Cr, Cd, Pb, Cu, Ni, B, Fe, Mn, and Zn pollution levels and the potential pollutant sources of these elements. Significantly higher heavy metal concentrations were observed in the middle and end parts of the lake compared to other regions. It can be said that agricultural drainage, coal mining activities, surface flows, wastewater treatment plants and old wild waste storage areas are effective in these regions.

**Table 8:** Index analysis results according to sampling points

No	Latitude	Longitude	WQI	HPI	HEI	HQ	RI	CPI
1	36,59,913	32,87,621	48	0.015508	0.001177	0.003984	0.004006	0.000147
2	36,59,827	32,85,623	46.81	0.011961	0.00114	0.003144	0.003227	0.000142
3	36,61,668	32,83,903	47.31	0.023576	0.001549	0.006297	0.005659	0.000194
4	36,57,398	32,7739	47.06	0.012117	0.001153	0.002956	0.003467	0.000144
5	36,58,567	32,84,476	45.8	0.019499	0.001404	0.005181	0.004639	0.000175
6	36,56,827	32,87,582	47.89	0.014225	0.00131	0.004677	0.003924	0.000164
7	36,55,106	32,90,298	46.42	0.011498	0.00111	0.004249	0.003195	0.000139
8	36,53,565	32,92,939	46.64	0.021887	0.001634	0.008356	0.005847	0.000201
9	36,51,081	32,9405	47.77	0.021533	0.001509	0.007648	0.005358	0.000185
10	36,54,644	32,94,718	47.23	0.014096	0.001086	0.008598	0.00323	0.000135
11	36,56,693	32,96,562	46.98	0.028434	0.001817	0.004281	0.006778	0.000215
12	36,58,017	32,91,965	47.94	0.015946	0.00141	0.005177	0.004499	0.000176
Average values			47.15	0.017523	0.001358	0.005379	0.004486	0.000168

Cd concentrations are concentrated in the eastern and southeastern regions. The maximum value was measured as 8.6 × 10^−5^ µg/L, which is well below the 5 µg/L limit specified in the TS 266 standard. This situation can be associated with the effects of the wastewater treatment plant located close to sampling point number 1, the coal mine and the old wild waste storage area located close to sampling point number 3. An increase in the distribution of Cr was observed in the central and eastern regions. The maximum value was 8.54 × 10^−4^ µg/L, which is lower than the TS 266 standard (50 µg/L). Potential reasons for higher concentrations in these regions include agricultural activities, surface flows and the mine site located close to sampling point number 3. (Fristachi & Choudhury, 2017; Olvera & Avelizapa, 2022; Teta et al., 2017; Yunusa et al., 2023; Zeng et al., 2020).

Cu concentrations are concentrated in the eastern and southeastern regions and recorded as a maximum of 0.0018 µg/L. Agricultural fertilizers, pesticides and mining sites are considered to be the main sources of this increase (Adrees et al., 2015; Dipti et al., 2023; Erdoğan et al., 2023; Kaymak, 2017; Shukla et al., 2024). It is well below the 2000 µg/L limit specified by the TS 266 standard. Fe distribution, especially in the eastern regions, is below the limit values, although it shows high density. The maximum value of 0.133 µg/L was recorded and this was associated with erosion, surface runoff and water sources from points 9 and 12 feeding the lake (Björnerås, 2019; Michalopoulou et al., 2024; Tometin et al., 2023; Wu et al., 2019). It is well below the 200 µg/L limit in the TS 266 standard.

An increase in Mn distribution was observed in the central and eastern parts. The maximum value was determined as 0.061 µg/L and it was found to be below the 50 µg/L limit specified in the TS 266 standard. This result can be explained by the effects of sediment transport, surface flows, the mining site near point 3 and the wild waste dump site (Håkanson, 1993; Hansel et al., 2002; Peng et al., 2016; Ito et al., 2008). Ni concentrations were concentrated in the western and eastern parts and were determined as a maximum of 0.0042 µg/L. Coal mining and old waste dumps can be considered as potential sources of this pollution (Buruah et al., 2022; Rizwan et al., 2024). It was observed that the 20 µg/L limit values according to the TS 266 standard were not exceeded.

The maximum value for Pb was measured as 0.0089 µg/L and it was observed to be concentrated in the southeastern region. It can be evaluated that wastewater treatment plant and agricultural drainage caused this concentration (Akhtar et al., 2022; Kassim et al., 2022; Vogt et al., 2019). It remained below the 10 µg/L limit specified according to TS 266 and WHO standards. Zn concentrations increase from the center to the east. The maximum was measured as 0.046 µg/L. Agricultural activities and surface runoff may have caused the Zn concentrations to increase (Bing et al., 2022; Junqueira et al., 2024; Zhao et al., 2022). It is very far from the 5000 µg/L limit specified in the TS 266 standard.

In general, heavy metal pollution is increasing especially in the eastern and southeastern regions. Agricultural drainage, coal mining, surface runoff, wastewater treatment plants and old wild waste dumps are prominent among the pollution sources. According to TS 266 and WHO standards, general water quality is at a safe level. However, localized pollution increase was observed in some local areas compared to other regions and this situation requires more detailed studies.

Index analysis results (Table 8) show that WQI values vary between 45.8 and 48.0 and water quality is generally in the "good" class. WQI results reveal that lake water is at acceptable levels even in regions affected by agricultural and mining activities. However, it is noteworthy that more pollution areas are formed in the central and southeastern regions compared to other regions. HPI values vary between 0.011 and 0.028, indicating a low level of pollution. HEI has a value of 0.001358 and is safe, HQ has a value of 0.005379 and is at an acceptable risk level, RI has a value of 0.004486 and is in the clean water category with a low ecological risk and CPI has a value of 0.000168. These indices show that heavy metal pollution in the lake is at a low level, water quality is at low risk, and negative effects on the ecosystem are minimal (Ateş et al., 2020; Biedunkova & Kuznietsov, 2024; Şener et al., 2023).

**Fig. 3:**
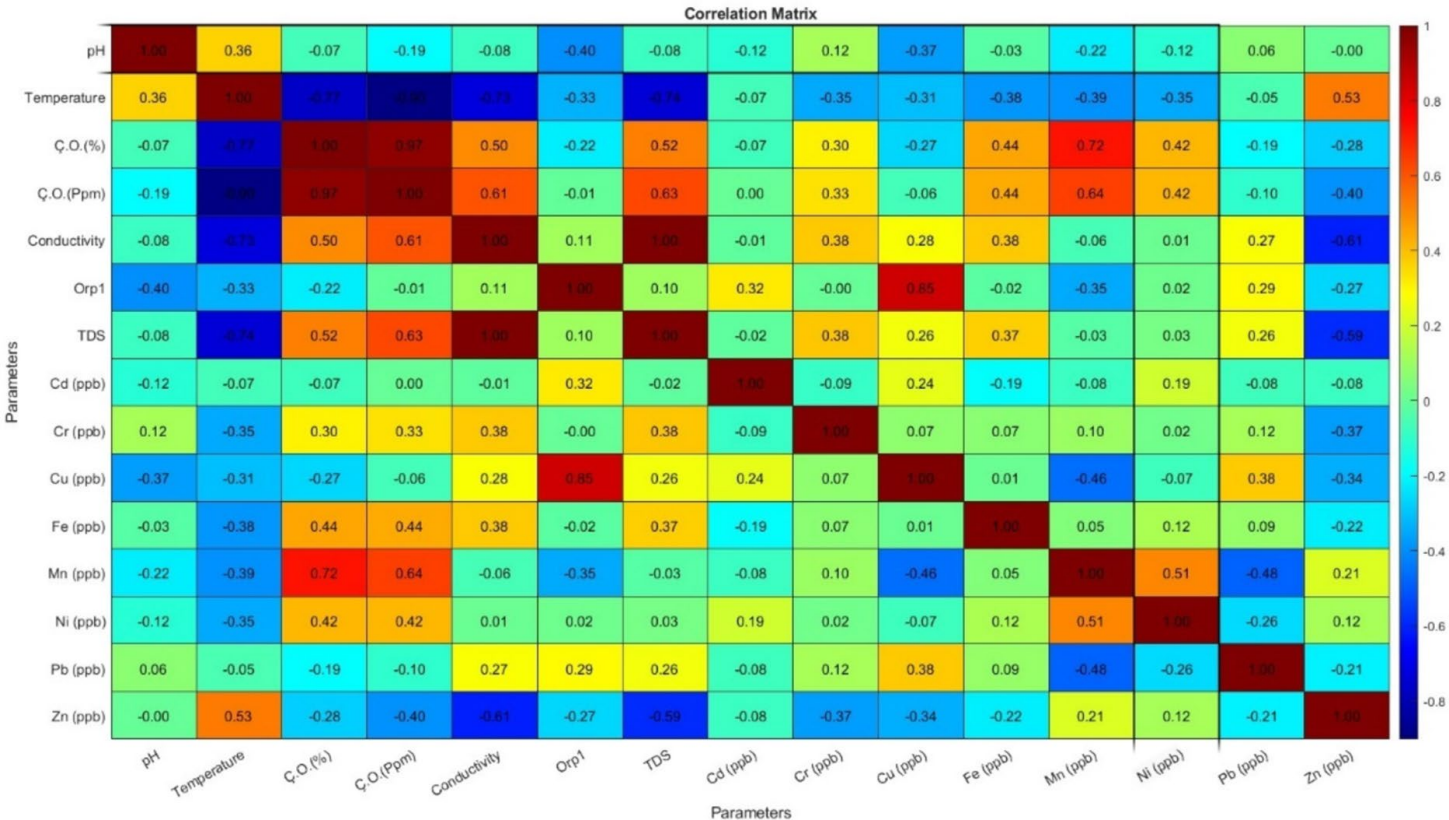
Correlation analysis of parameters

Using correlation analysis (Fig. 3), the relationships between water quality parameters and heavy metal concentrations were investigated in detail. Negative correlations were detected between pH values and Cd, Cu, Fe, Mn, Ni and Zn. Correlations were observed between Cd and pH at − 0.12, Cu with − 0.37, Fe with − 0.03, Mn with − 0.22, Ni with − 0.12 and Zn with − 0.006. This shows that with increasing pH values, the solubility of these metals decreases and their precipitation tendency increases (Nath et al., 2024; Zou et al., 2024). Especially the negative correlation of pH with Cu and Mn indicates that these metals may decrease on the water surface by oxidation and precipitation mechanisms (Zou et al., 2024). In addition, a weak positive correlation of 0.12 between Cr and pH and 0.06 between Pb and pH reveals that Cr is affected by pH variation and Pb is less affected by pH changes (Stanescu et al., 2024).

Strong negative correlations were observed between temperature and conductivity (− 0.73), TDS (− 0.74), and DO (− 0.77). This shows that increasing temperature decreases the amount of Do, the amount of dissolved solids and conductivity (Adjovu et al., 2023). In addition, moderate negative correlations were found between temperature and all heavy metals except Zn. This result suggests that increasing temperature may decrease the concentrations of these metals in water by increasing their tendency to precipitate (Liao et al., 2023).

Negative correlations were observed between DO and Cd (− 0.01), Cu (− 0.06), Pb (− 0.10) and Zn (− 0.40). The negative correlation shows that especially Pb and Zn tend to precipitate more. In addition, positive correlations were found between DO and Cr (0.33), Fe (0.44), Mn (0.64) and Ni (0.42). The positive correlation suggests that these heavy metals may have the potential to remain in dissolved form in oxygenated environments (Huang et al., 2024; Lin et al., 2024; Zhang et al., 2024).

A correlation close to 1 was found between conductivity and TDS. A negative correlation was observed between conductivity and TDS with Cd (− 0.01), Mn (− 0.06) and Zn (− 0.61). This correlation, especially the strong negative correlation of Zn, indicates that the solubility of zinc ions is low or tends to precipitate (da Silva et al., 2024; Liu et al., 2024c). A positive correlation was observed between conductivity and TDS with Cr (0.38), Cu (0.28), Fe (0.38), Ni (0.01) and Pb (0.27). The positive correlation indicates that these heavy metals tend to be found in dissolved forms in water and contribute to the ionic charge distribution (Tereshchenko et al., 2024).

When the relationships between heavy metals in lake water were examined, a positive correlation of 0.24 was observed between Cd and Cu. This indicates that Cd and Cu may come from a common source (e.g. industrial wastes or agricultural inputs). A positive correlation of 0.51 was found between Mn and Ni and 0.21 between Mn and Zn, indicating that these metals have similar transport and precipitation processes. A negative relationship of − 0.48 was observed between Mn and Pb and − 0.46 between Cu, while a positive relationship of 0.38 was found between Cu and Pb. The relationship between Mn, Pb and Cu indicates that Pb and Cu have similar transport and precipitation processes, and Mn may be affected by different sources or the precipitation and transport mechanism may be different (Chang et al., 2022; Wang et al., 2023).

**Fig. 4:**
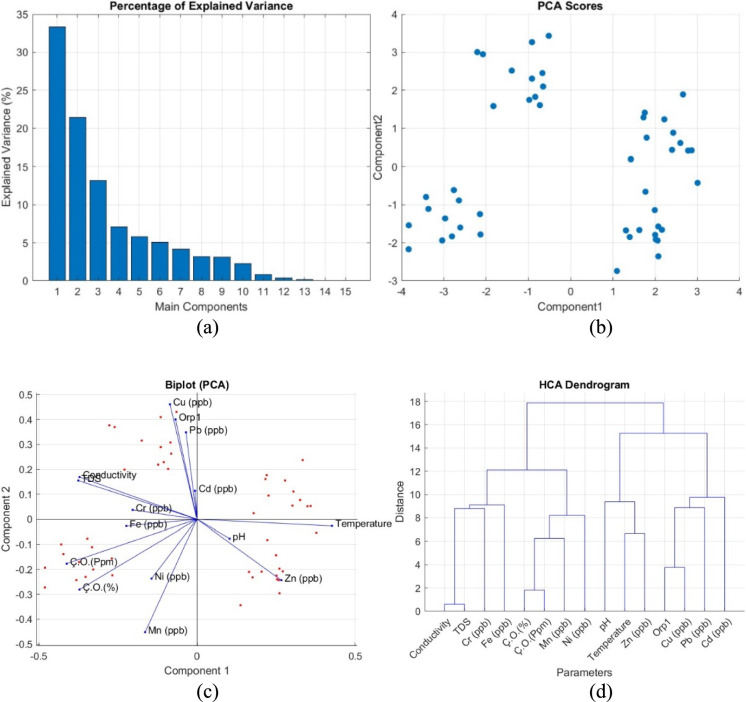
Component Analysis of Parameters; **a** Percentage of Explained Variance, **b** PCA Scores Graph, **c** Biplot PCA Graph, **d** Hierarchical Cluster Analysis (HCA) dendrogram

According to the PCA explained variance percentage graph of heavy metals in lake water (Fig. 4a), the first component has an explanatory power of 34%, the second component 22% and the third component 14%. The first and second principal components explain 56% of the total variance. This shows that a large part of the analyzed variables can be represented by two main components. The high explanation percentage of the first component increases the possibility that especially heavy metals are affected by common sources (Jiang et al., 2013; Zhou et al., 2008). The PCA score graph (Fig. 4b) shows that the data are divided into 3 different clusters. This indicates that the samples may have been affected by different sources. The first cluster includes samples with low pollution, while the second and third clusters include samples with high concentrations. This distribution indicates different effects and sources, with three clusters on the lake (Zhou et al., 2008; Liu & Zhang, 2010).

HCA (Fig. 4c) visualized the similarities between the parameters. In the dendrogram analysis, it is seen that conductivity and TDS form a group close to each other, Cr and Fe show similar properties, Mn and Ni are close to each other and show similar properties. These groupings support that the parameters may have common sources or similar transport mechanisms. In addition, the fact that Cu, Pb and Cd are close to each other and show similar properties and cluster differently from other heavy metals reveals that these metals may have been affected by different sources than other heavy metals (Jung et al., 2024).

The Biplot PCA plot for heavy metals in lake water (Fig. 4d) shows the orientations and charges of the variables on the main components. Zn is located in the direction of temperature and pH and shows a positive relationship with these parameters. This shows that the solubility of Zn is sensitive to temperature and pH levels. On the other hand, Mn and Ni are located on the negative axis and indicate that these metals are inversely related to temperature and pH. Pb, Cu, Cd and ORP are aligned in the same direction, suggesting that these parameters are highly correlated with each other and are affected by a common source, possibly indicating anthropogenic sources (industrial wastes, mining or agricultural activities). Conductivity, TDS and Cr are located in the same direction and are associated with transport and solubility processes depending on the dissolved ion density. It was observed that Cr tends to remain in dissolved form at high conductivity and TDS levels. This situation reveals that chromium pollution may be more intense in regions where dissolved salts and ions are present. Fe, Mn, Ni and Ç.O exhibit similar trends, indicating that they are affected by oxidation and precipitation mechanisms. It is observed that Mn and Ni tend to precipitate depending on oxygen levels, while Fe is directly affected by dissolved oxygen levels. PCA results are also consistent with correlation analysis and HCA analysis results and support the findings. These results reveal how the solubility and transport dynamics of heavy metals are affected by environmental parameters. Similar studies in the literature are consistent with our findings (Ateş et al., 2020; Chen et al., 2022; Iannucci, 2021; Li et al., 2020; Wu et al., 2017).

In general, correlation analyses, PCA and HCA results support each other and reveal that the main factors affecting water quality (pH, temperature, conductivity, DO, TDS) are closely related to heavy metal concentrations. These results show that heavy metal pollution in the region is closely related to geochemical processes and anthropogenic activities. Correlation and clustering results are also consistent with previous studies in the literature and provide an important basis for the creation of water quality management plans (Ateş et al., 2020; Hayder et al., 2022; Hazarika & Kalita, 2020; Jung et al., 2024; Kareem & Leventeli, 2024; Zhou et al., 2008;).

## Conclusion

In this study, heavy metal pollution and water quality in Ermenek Dam Lake were evaluated. The findings show that the water quality is generally in accordance with TS 266 and WHO standards and at safe levels. The concentrations of the examined heavy metals (Cd, Cr, Cu, Fe, Mn, Ni, Pb and Zn) remained well below the determined limit values. Pollution analyses revealed that heavy metal concentrations were relatively higher in the eastern and southeastern regions. This situation can be explained by the effects originating from agricultural drainage, coal mining, surface flows, wastewater treatment plant and old wild waste storage areas. However, these increases are still considered safe according to the standards. Index analysis results show that the water is generally in the 'good' quality class. WQI values were found to be at acceptable levels, varying between 45.8 and 48.0. HPI, HEI, HQ, RI and CPI analysis results also show that heavy metal pollution is at low levels, ecological risk remains at a minimal level and lake water can be evaluated as a safe drinking water source.

Correlation analyses detailed the relationships between pH, temperature, conductivity, dissolved oxygen (DO), total dissolved solids (TDS) and heavy metals. The negative correlation of pH with some heavy metals revealed that solubility and precipitation dynamics were affected by pH changes. Strong negative correlations between temperature and DO, TDS and conductivity indicated that increasing temperature decreased dissolved substances and oxygen levels. In addition, precipitation and solubility processes of heavy metals were affected by environmental factors. PCA and HCA analyses provided important information in determining the sources and distributions of heavy metals. According to PCA results, the first two principal components explained 56% of the total variance and the possibility of the parameters being affected by common sources was revealed. HCA dendrogram analyses supported the conclusion that they could be affected by common sources by visualizing the similarities and groupings of the parameters. Spatial distribution maps confirmed that the pollution in the lake is localized under the influence of agricultural drainage, coal mining, surface runoff and wastewater treatment plants. It is seen that the regions where heavy metals are concentrated vary according to heavy metal groups. This confirms the PCA and HCA results.

These findings indicate that Ermenek Dam Lake is generally low risk, but localized pollution may have long-term effects. In particular, agricultural and mining activities and wastewater treatment plants should be kept under control, which is critical for the sustainability of the lake ecosystem. In addition, monitoring the long-term effects of metals such as Cd, Pb and Zn and evaluating sediment–water interactions are recommended.

## Data Availability

No datasets were generated or analysed during the current study.
